# Factors influencing the results of air samplers in closed, air-conditioned patient rooms—a numerical study

**DOI:** 10.1007/s11356-025-36036-9

**Published:** 2025-03-05

**Authors:** Christian Ernst, Martin Heinrich, Rüdiger Schwarze

**Affiliations:** Institute for Mechanics and Fluid Dynamics, TU Bergakademie Freiberg, Lampadiusstraße 4, Freiberg, 09599 Saxony Germany

**Keywords:** Aerosol distribution, Air sampling, SARS-CoV-2, CFD, ILES, Patient room, Droplet evaporation

## Abstract

Several air sampling studies have been conducted to assess the risk of airborne transmission since the outbreak of SARS-CoV-2 in late 2019. However, differences in sampler positioning and positive collection results in more distant locations suggest an interaction between the sampler and the indoor air flow, altering the aerosol distribution. This study aims to investigate this influence by studying multiple collector positions, patient exhalation modes, and two ventilation setups in a 3D simulation model using implicit large-eddy simulations along with evaporative Lagrangian particles. The results of this study show a higher total aerosol particle amount in the patient room with the deployment of a sampling device, possibly increasing infection risk for medical personal during and shortly after a sampling procedure. Furthermore, the variation of the collector position reveals an impact on the sampling yield, thus rendering even more distant positions viable and potentially beneficial in terms of maintaining sampler performance and increasing patient comfort. Moreover, the influence of ventilation was investigated suggesting the deactivation to increase aerosol concentration during sampling campaigns for efficient sampling. Additionally, results indicate an impact on room flow by air samplers and subsequent sampling yield, potentially necessitating reassessments of conclusions drawn from previous sampler studies. Finally, it can be concluded that future air sampling campaigns, which are preliminarily assessed using numerical simulation, could benefit from advantageous positioning to aid sampling success.

## Introduction

With the wide spread of SARS-CoV-2 several transmission routes are discussed among which airborne transmission is the most prevalent. Therefore, several studies focused on the evidence of viral material in the air. An air sampling study in 2020 by Liu et al. (2020) managed to sample viral ribonucleic acid (RNA) in two Wuhan hospitals and proposed the possibility of aerosol infection. Following studies with often similar arrangements among each other regarding the sampler position, sampler inflow rate, and air exchanges per hour (ACH) in the room deliver contrary results. Here, Cheng et al. (2020) and Ayuso et al. (2022) conducted a similar sampling campaign while only Ayuso et al. (2022) found viral material. Moreover, Kim et al. (2020) and da Silva et al. (2022) also found conflicting results with similar setups. It is assumed by da Silva et al. (2022) that the intubation of a patient functioned as a high viral release event. However, Schoen et al. (2022) could not provide evidence of viral material during active labor, another potential high viral release event.

Several air sampling studies by Chia et al. (2020); Ang et al. (2022); Wilson et al. (2022); Razzini et al. (2020); Santarpia et al. (2020) and their findings of viral RNA in more remote places (Santarpia et al. 2020; Wilson et al. 2022) and with different sampler positions (Chia et al. 2020; Ang et al. 2022) imply an interaction between sampling device, room flow, ventilation system, and resulting sampler yield.

This dynamic system of air flow and aerosol spread within ventilated, confined spaces is frequently investigated with computational fluid dynamics (CFD) tools in recent years. Here, detailed studies regarding the aerosol distribution during breathing and speaking are investigated by Gupta et al. (2011) for an airliner cabin, during passenger rides on airport vehicles by Zhu et al. (2022), and for the effect of air conditioning in elevators by Dbouk and Drikakis (2021) and Nouri et al. (2021).

Furthermore, the traditional CFD approach is extended by Ghoroghi et al. (2022) by coupling it with transmission models and the Discrete Event Simulation method while the Lattice-Boltzmann-Method is employed by Beaussier et al. (2022) to evaluate the risk on a hospital floor based on the water vapor concentration.

Noteworthy is the validation effort of Reynolds-averaged Navier–Stokes (RANS) simulation with oral bacteria (Li et al. 2022) and different exhalation modes based on the large eddy simulation (LES) approach (Zhang et al. 2019) with regards to the aerosol dispersion while droplet evaporation is neglected. The evaporation is accounted for by Vuorinen et al. (2020) and Dao and Kim (2022) providing evidence that evaporated particles are longer airborne (Dao and Kim 2022).

Moreover, CFD studies conducted by Huang et al. (2022) and Saw et al. (2021) specifically addressing the aerosol distribution within a hospital bed room complementing air sampling campaigns. Thereby, transient RANS simulations with $k -\epsilon$ turbulence models are employed to investigate the spread of Lagrangian, single-component particles with a diameter range of 70 nm to 10 µm (Saw et al. 2021) and a uniform diameter of 0.4 µm (Huang et al. 2022). The non-evaporative particles are induced by different exhalation modes like breathing, speaking, and coughing with constant outlet velocities while the impact of the sampling devices on the air flow is omitted in these studies.

However, it is assumed that the interaction between the sampler and room flow alters the aerosol distribution within the patient’s room, thus, influencing the sampling results. This assumption is further underlined by the different results even for similar studies indicating other factors impact on the sampling process.

Therefore, the aim of this study^1^ is to address the influence of the operating sampler and its position on the sampler yield as well as the room air flow which is assumed to have impacted similar studies in the literature. In addition, previously conducted numerical investigations are extended by considering a sampler model and its influence along with the evaporative nature of exhaled droplets emitted by different exhalation modes with more realistic injection functions.

For this reason, the influence of the collector position is investigated as well as the effect of the ventilation system on the sampling campaign based on simulations with a numerical model using an implicit large-eddy simulation (ILES) approach and an experimental particle size distribution (PSD) from literature.

## Numerical model

### A. governing equations

The numerical model is divided into a continuous air phase and a solid, dispersed particle phase consisting of evaporative aerosol particles. The governing equations of the air flow are mass

1 $$\begin{aligned} \nabla \cdot \pmb {U} = 0, \end{aligned}$$

momentum

2 $$\begin{aligned} \frac{\partial \rho _{0} \pmb {U}}{\partial t} + \nabla \cdot \left( \rho _{0} \pmb {U} \pmb {U} \right) = - \nabla p + \nabla \cdot \left( \eta \nabla \pmb {U} \right) + \pmb {g} \rho (T) \end{aligned}$$

and energy

3 $$\begin{aligned} \frac{\partial T}{\partial t} + \nabla \cdot \left( \pmb {U}T \right) = \alpha \nabla ^{2} T, \end{aligned}$$

with the velocity vector $\pmb {U}$, pressure *p*, gravitational acceleration $\pmb {g}$, temperature *T* and the reference density $\rho _{0}$, thermal diffusivity $\alpha$, and dynamic viscosity $\eta$ as material properties of the air.

Density variations due to temperature differences are considered only in the buoyancy term with the *Boussinesq* approximation

4 $$\begin{aligned} \rho = \rho _{0} \left[ 1 - \beta \left( T - T_{0} \right) \right] , \end{aligned}$$

which is valid in the range of

5 $$\begin{aligned} \frac{\beta \left( T - T_{0} \right) }{\rho _{0}}<< 1, \end{aligned}$$

with $$\beta = 3 \times 10^{-3}\ \textrm{K}^{-1}$$, $$T_{0} = 300\ \textrm{K}$$ and $$\rho _{0} = 1.225\ \textrm{kg}\ \textrm{m}^{-3}$$.

**Fig. 1:**
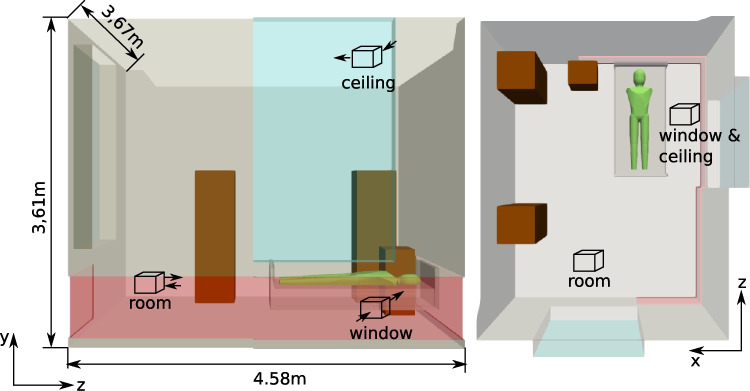
Side and top view of computational domain with dimensions and schematic sampler positions and in-/outtake orientation

Turbulence is partially resolved using the ILES approach (Fureby and Grinstein 2002) and Eqs. 3–6 are solved using the open-source CFD library *OpenFOAM v2112* (Weller et al. 1998).

The movement of each particle of the aerosol phase is governed by their equation of motion

6 $$\begin{aligned} \rho \cdot V(r) \cdot \frac{d \pmb {U}_p}{d t} = \pmb {F}_G + \pmb {F}_D + \pmb {F}_P + \pmb {F}_B, \end{aligned}$$

with the particle density $\rho$ calculated as seen by Rosti et al. (2020), the particle volume *V*, the particles current radius *r*, the particle velocity vector $\pmb {U}_{p}$ and the corresponding forces for gravity $\pmb {F}_{G}$, drag $\pmb {F}_{D}$, pressure $\pmb {F}_{P}$, and buoyancy $\pmb {F}_{B}$.

### B. droplet evaporation

To account for the evaporation of the volatile droplet components the *bouyantBoussinesqPimpleFoam* solver is modified according to the implementation of Rosti et al. (2020). Therefore, a relative humidity $h_{rel}$ field is added to the air flow (Celani et al. 2005) which is governed by

7 $$\begin{aligned} \frac{\partial h_{rel}}{\partial t} + \nabla \cdot \left( \pmb {U} h_{rel} \right) = D_{V} \Delta h_{rel}, \end{aligned}$$

with the diffusivity of water vapor in air $D_{V} = 2.5 \times 10^{-6}\ \textrm{m}^{2}\textrm{s}^{-1}$ (Rosti et al. 2020). Furthermore, the change of the spherical droplets radius $\Delta r$ is influenced by $h_{rel}$ and can be described as (Pruppacher and Klett 2010)

8 $$\begin{aligned} \Delta r = \frac{\Delta t}{2 r} \cdot C_{R} \cdot \left( \left( h_{rel} \right) - e^{\frac{A}{r} - B \frac{R^3}{r^3 - R^3}} \right) , \end{aligned}$$

with the current radius of the droplet *r*, the time step $\Delta t$, the condensational growth rate $C_{R}$, and the radius of the droplet when totally dry *R*. The following modeling is conducted according to the supplementary information by Rosti et al. (2020). However, the size ratio between the fully dried particle and its initialization radius $r_{0}$ is assumed to be

9 $$\begin{aligned} \frac{R}{r_{0}} = {0.4} \end{aligned}$$

for the investigated relative humidity $h_{rel}$ of ${50\,\mathrm{\%}}$ (Marr et al. 2019) to account for the importance of proteins in aerosol particles according to Wang et al. (2023). The evaporated water does not contribute to the relative humidity $h_{rel}$ of air in the computational domain.

## Simulation setup

### A. computational domain and discretization

The domain is based on a real patients room in the St. Georg Hospital, Leipzig with a patient lying on the bed and facing the ceiling. In Fig. 1, the computational domain along with a schematic of the different sampler positions (*room*, *window*, and *ceiling*) and the orientation of the in- and outtakes is displayed in side and top view. The *window* position is chosen due to a real-world sampling position during air sampling studies at the St.Georg hospital while the *ceiling* position is chosen to observe the impact of height on the sampling results. The *room* site complements the effort to investigate a position further away from the patient.

**Fig. 2:**
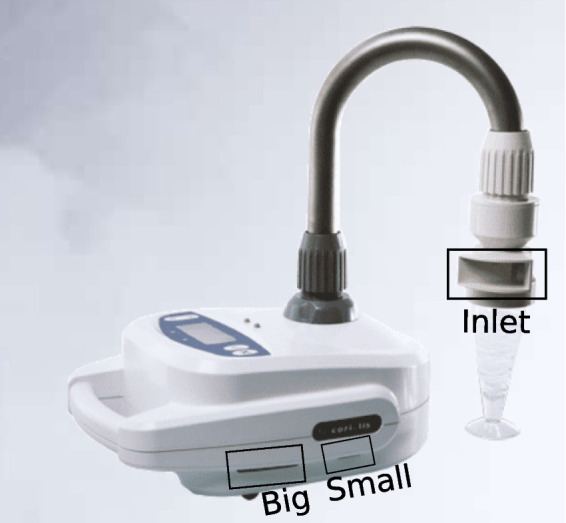
Simplified intake model from “Coriolis Micro” air sampler by “Bertin Technologies”

In Table 1, the center points of the sampling positions are listed while a simplified cuboid sampler geometry is shown in Fig. 2. For simplicity reasons only the sampler *Inlet* along with the *Big* and *Small* outlet are considered while the rest of the device is omitted. Both outlets feature a height of 10 mm while the *Big* outlet is 50 mm wide and the *Small* outlet has a width of 25 mm. The *Inlet* is 50 mm wide and 30 mm high.

**Table 1:** Position of sampler intakes center points in the patient room

	Center points		
Name	*x*	*y*	*z*
Room	2.55 m	0.82 m	1.00 m
Window	0.48 m	0.36 m	3.51 m
Ceiling	0.48 m	2.74 m	3.51 m

Based on the computational domain a hexa-hedral, structured mesh is created with the OpenFOAM tool *cfMesh*. The mesh features a minimum cell size of 0.5 mm near the patient, a maximum cell size of 40 mm, and a refined cell size of 10 to 20 mm near the walls leading to a total cell amount of about 3.3$\times 10^{6}$ in the domain.

The governing equations for the air flow are discretized using only second-order schemes as shown in Table 2. The flow field is solved employing the Pressure Implicit with the splitting of the operator for pressure-linked equations (PIMPLE) algorithm and the maximum Courant number $Co_{max}$ is ${5}$ while the mean Courant number $Co_{mean}$ is around 0.004. The simulations are calculated on the *Compute Cluster 2019* of the *TU Bergakademie Freiberg* using 96 Intel Xeon Cascade Lake-X Gold 6248 CPU cores with a base clock of 2.5 GHz and around 344 TFlops/s peak performance. Calculation time ranges from about 194 to 489 h.

**Table 2:** Numerical discretization schemes

Discretization	Term	Scheme
Time	$\frac{\partial }{\partial t}$	Backward
Gradient	$\nabla \pmb {U}$	Gaussian central differencing
	$$\nabla p$$	
Divergence	$\nabla \cdot (\pmb {U} \pmb {U})$	Gaussian linear upwind differencing
	$$\nabla \cdot (\pmb {U} T)$$	
	$$\nabla \cdot (\pmb {U} h_{rel})$$	
Laplace	$\nabla \cdot (\eta \nabla \pmb {U})$	Gaussian central differencing with a weighting factor $\psi = 0.8$

### B. boundary conditions

**Fig. 3:**
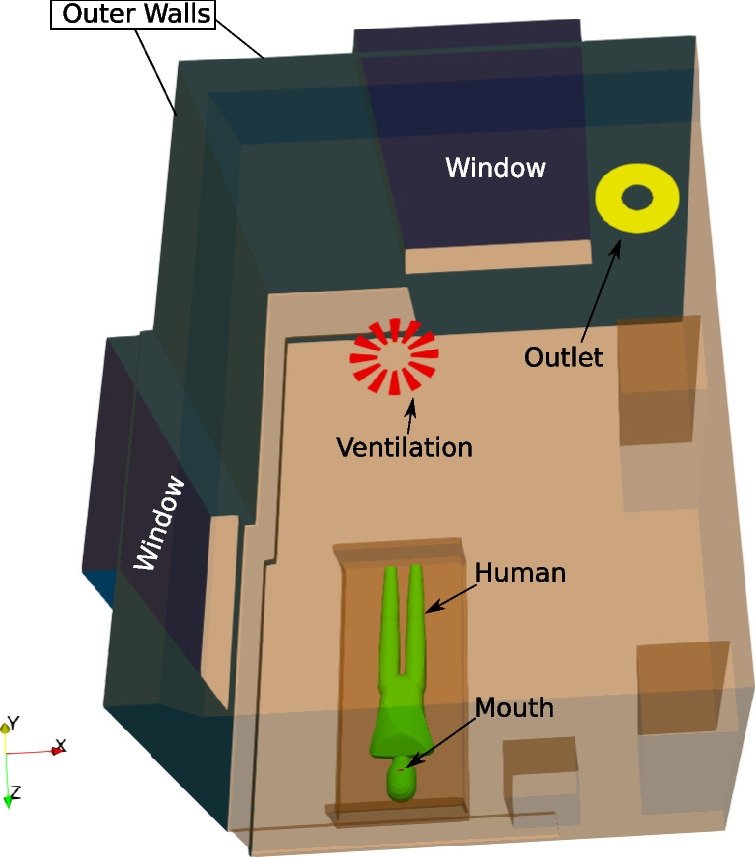
Computational domain with main boundaries

The computational domain with the main boundaries is displayed in Fig. 3. Here, the *Ventilation* boundary acts as an active air flow inlet if the heating, ventilation, and air conditioning (HVAC) system is activated and the *Outlet* boundary is a simple, passive air outlet. While the ventilation system is active the *Ventilation* boundary provides an air inflow flux of about $119\ \textrm{L} \textrm{s}^{-1}$ leading to an ACH of about $8.5\ \textrm{h}^{-1} =0.142\ \textrm{min}^{-1}$. The ventilation settings of the HVAC system are based on measurements at the St. Georg Hospital, Leipzig. For comparison, a setting with a deactivated HVAC system is investigated, too. Here, the *Ventilation* boundary becomes identical to the *Outlet* boundary and does not provide any air inflow.

The *Mouth* boundary inserts droplet particles depending on the investigated exhalation mode. For the patient, three different cases of activity are analyzed (i) pure *mouth breathing*, (ii) *speaking*, and (iii) *singing*.^2^ An overview of the investigated cases is provided in Table 3.

The velocity condition of the exhalation modes *mouth breathing* and *speaking* is simulated using a sine function with a periodic length *P* of 4 s and an amplitude *a* of $1.5\ \textrm{m}\ \textrm{s}^{-1}$ while the *singing* activity is calculated with a constant velocity *U* of $0.26\ \textrm{m}\ \textrm{s}^{-1}$.

The sampler model features an inflow volume flux $Q_{in}$ of $300\ \textrm{l}/\textrm{min}$ and two outflow volume fluxes $Q_{\text {Big}/\text {Small}}$ of $200\ \textrm{l}/\textrm{min}$ and $100\ \textrm{l}/\textrm{min}$, respectively. A constant relative air humidity $h_{rel}$ of ${50\,\mathrm{\%}}$ is assumed in the domain. The sampling time is $t_{\text {sampling}}$ is ${10\,\mathrm{\min }}$. In addition, all boundary conditions are listed in Table 5.

**Table 3:** Investigated simulation cases

Sampler position	Ventilation	Exhalation mode	Sampler active?
Room	Off	Singing	Active
	On	Singing	Active
			Inactive
		Speaking	Active
			Inactive
		Breathing	Active
			Inactive
Ceiling	On	Singing	Active
Window

**Table 4:** Particle exhalation rates and PSD for the exhalation modes *mouth breathing*, *speaking*, and *singing* by Archer et al. (2022)

	Mouth breathing	Speaking	Singing
Exhalation rates $\left[ \textrm{s}^{-1} \right]$	10	60	200
Initial diameter $d_{0} [$µm]	Probability $[\%]$		
${1.5}$	${55.58}$	${46.48}$	${35.93}$
${2.4}$	${27.79}$	${23.24}$	${23.10}$
${3.7}$	${12.51}$	${11.62}$	${15.40}$
${4.9}$	${2.78}$	${8.71}$	${12.83}$
${6.1}$	${0.56}$	${4.07}$	${5.10}$
${7.3}$	${0.42}$	${2.90}$	${5.10}$
${8.5}$	${0.17}$	${1.16}$	${1.28}$
${9.8}$	${0.10}$	${0.87}$	${0.77}$
${11.0}$	${0.10}$	${0.52}$	${0.18}$
${12.2}$	−	${0.23}$	${0.15}$
${13.4}$	${0.04}$	${0.15}$	${0.05}$
${14.6}$	−	−	${0.03}$
${15.9}$	−	${0.04}$	${0.01}$
$$ {17.1} $$	−	−	$$< {0.01} $$

In Table 4, the PSD along with the exhalation rates for each exhalation mode are listed for the wet, initial state of the droplet-based on a size ratio $\frac{R}{r_0}$ of ${0.4}$ at an assumed relative humidity of ${50\,\mathrm{\%}}$ (Marr et al. 2019) in the patient room (Table 5).

To model the function of the sampler, aerosol particles are counted and deleted from the simulation when they pass the *Inlet* interface of the sampler in the computational domain.

## Results

**Fig. 4:**
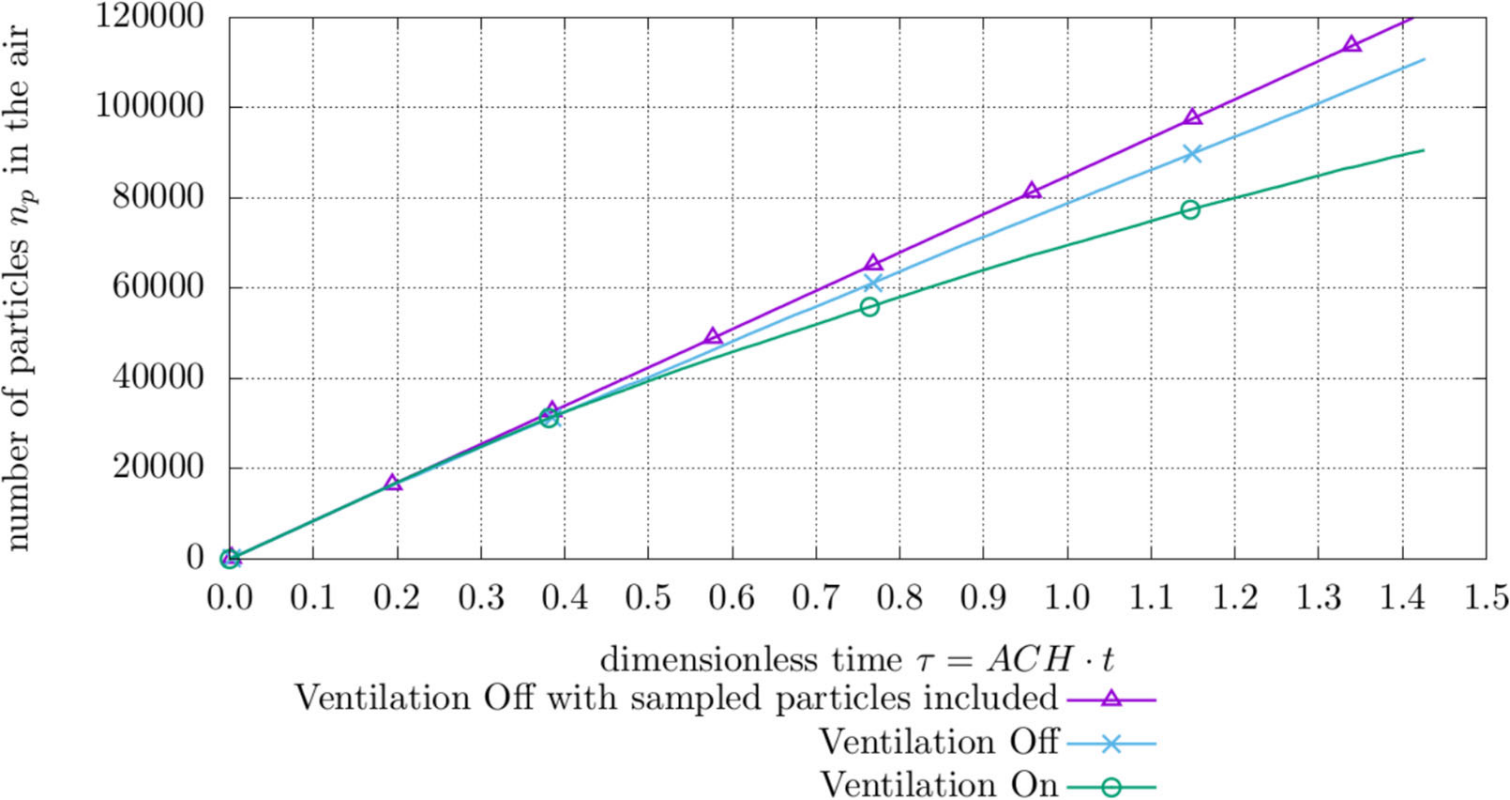
Amount of total particles $n_{p}$ in the hospital room with different ventilation settings based on the *room* site with *singing* exhalation mode

**Fig. 5:**
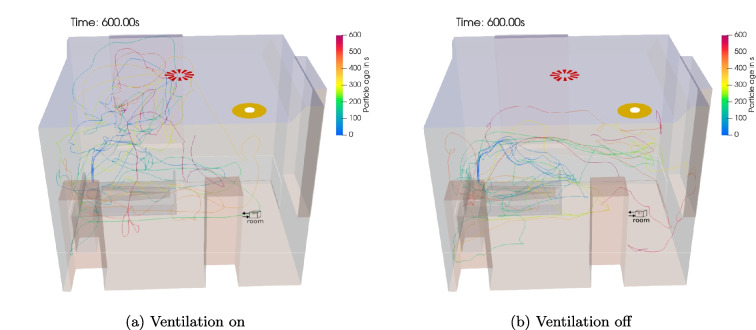
Particle pathlines while *singing* and a collector at the *room* site with HVAC system switched **a** on and **b** off

Figures 4 and 5 visualize the impact of the HVAC system on the particle behavior. Figure 4 shows how the total number of aerosol particles $n_p$ in the patient room develops over dimensionless time $\tau = \text {ACH} \cdot t$ with active or inactive HVAC and an active sampler at the *room* site while the patient is *singing*. As expected, the HVAC system switched off leads to an increased amount of particles in the domain while the plot featuring an active HVAC system shows less particles in the system and a decrease in growth over time.

**Table 5:** Boundary conditions for computational domain

Boundary	Temperature *T*	Velocity *U*	Pressure *p*
Mouth	$T= 310\ \textrm{K}$	$U_{breath,speak} = a \cdot \sin (\frac{2 \pi }{P} \cdot t)$	Ambient
		$$U_{sing}=0.26\ \textrm{m}\ \textrm{s}^{-1}$$	
Human	$T= 293\ \textrm{K}$	$U=0\ \textrm{m}\ \textrm{s}^{-1}$	$\nabla p = {0}$
Outlet	$\nabla T_{outflow} = 0$, $T_{inflow}= 291\ \textrm{K}$	$\nabla U = {0}$	Ambient
Ventilation	Ventilation on		
	$T_{vent.}= 296\ \textrm{K}$	$Q = 119\ \textrm{L}\ \textrm{s}^{-1}$	$\nabla p = {0}$
	Ventilation off		
	$\nabla T_{outflow}= {0}$, $T_{inflow}= 291\ \textrm{K}$	$\nabla U = {0}$	Ambient
Windows & Outer Walls	$T= 291\ \textrm{K}$	$U= 0\ \textrm{m}\ \textrm{s}^{-1}$	$\nabla p = {0}$
Inlet	$$\nabla T_{outflow}$$ $$= {0} $$, $$T_{inflow}$$ $$= 291\ \textrm{K}$$	$$Q_{in}= 300\ \textrm{l}/\textrm{min}$$	Ambient
Big		$Q_{\text {Big}}= 200\ \textrm{l}/\textrm{min}$	
Small		$Q_{\text {Small}}= 100\ \textrm{l}/\textrm{min}$	
Others	$\nabla T = {0}$	$U= 0\ \textrm{m}\ \textrm{s}^{-1}$	$\nabla p = {0}$

**Fig. 6:**
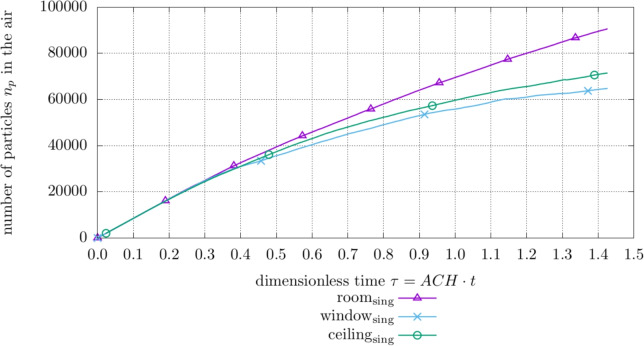
Amount of total particles $n_{p}$ in the hospital room for different sampling sites with active HVAC system and a *singing* patient

**Fig. 7:**
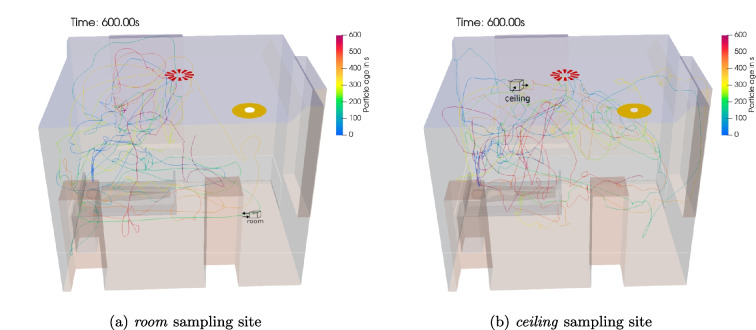
Particle pathlines while *singing* with the active HVAC system and a sampler at the **a** *room* and **b** *ceiling* site

**Fig. 8:**
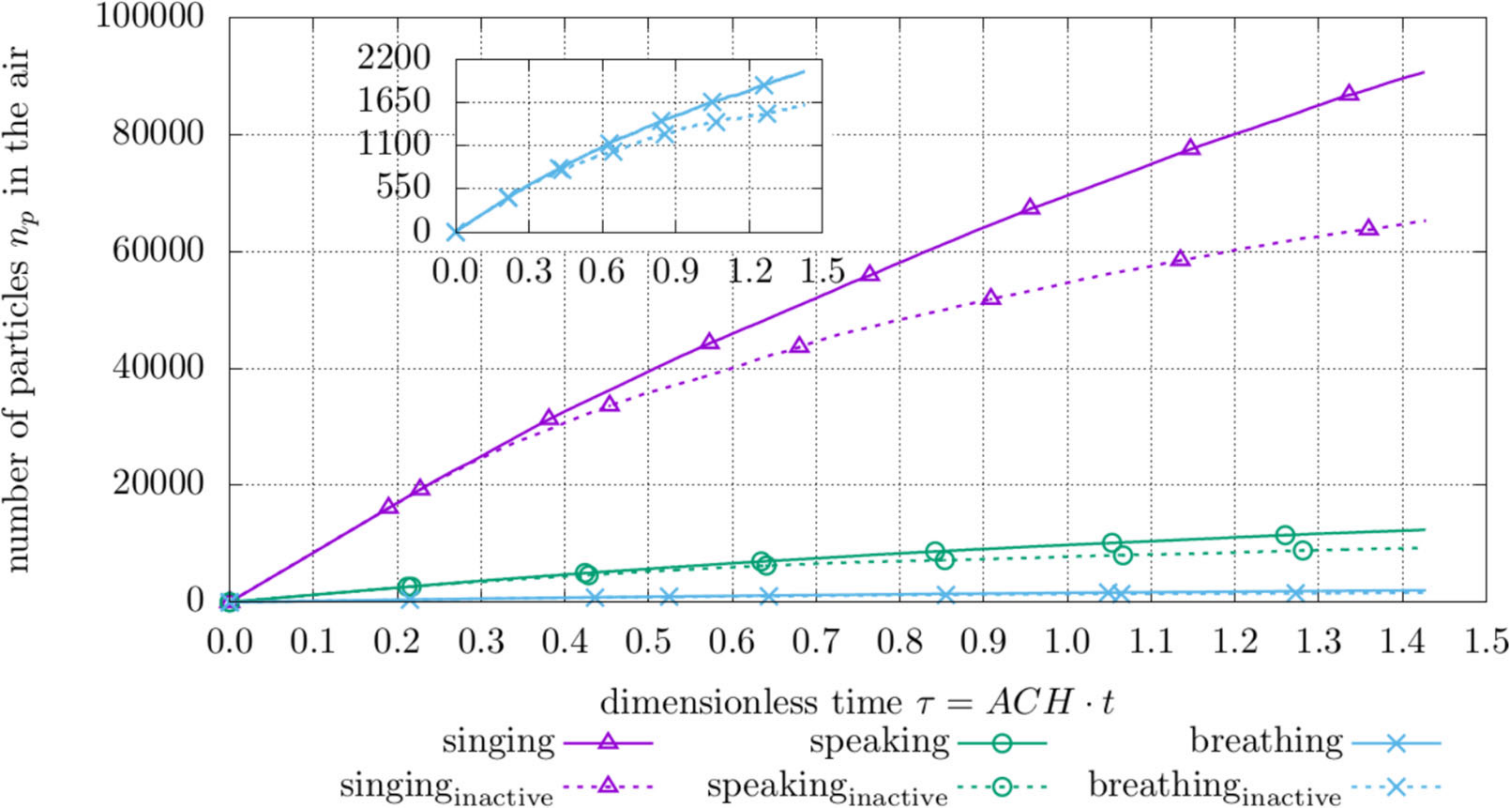
Amount of total particles $n_{p}$ in the hospital room with different exhalation modes and (in-)active samplers for the *room* site with active HVAC system

Figure 5 shows 5 example trajectories of aerosol particles in the room air flow with the HVAC system switched on and off. The particle trajectories show how the aerosol particles exhaled at the patient’s mouth move in the room in 10 min. Due to the turbulence of the room airflow, all trajectories show random walk behavior. In the case of the HVAC system being turned on, the fresh air jet emanating from the ventilation inlet crucially affects the particle behavior. The paths tend to show a vertical recirculating motion of the particles around this jet. When the HVAC system is switched off, on the other hand, the particle paths tend to run horizontally. The corresponding room air flow is induced here by the active collector.

The effect of different sampling sites with the active HVAC system and a *singing* patient on the particle amount $n_p$ is shown in Fig. 6. Noteworthy is the influence of the sampler position on the total amount of particles in the system. The *room* position leads to the highest total particle amount while the *ceiling* and *window* positions are close to each other, however, considerably below the particle amount with the *room* collection site.

Figure 7 shows 5 exemplary particle pathlines for the *room* and *ceiling* site to visualize the aerosol particle behavior. It can be observed that the particle paths have a tendency to move to the *Outlet* due to the *room* samplers influence, thus, leading to significant less aerosol particles leaving the domain. Moreover, a sampler-induced circulation of particles can be observed. The increased amount of particles in the patient room in addition to the occurring circulation of particles explains the good sampler performance this far from the emitting patient. In contrast, the particle paths with the *ceiling* sampler show that particles mainly travel in the upper domain half closer to the *Outlet* boundary leading to an increased amount of escaping particles compared to the *room* site.

Figure 8 highlights the effect of an (in-)active sampling device and different exhalation modes on the overall aerosol particle amount in the room with a *room* collection site and active HVAC. It is observed that the employment of a sampler, compared to a configuration featuring no sampling device at all, counter-intuitively leads to a higher total particle amount in the system. This presumably benefits sampler yield as well as elevating the infection risk for medical staff during and shortly after the sampling procedure. In addition, the difference in total particle amount between *active* and *inactive* sampler slightly increases over time while higher aerosol emission rates substantially leverage the disparity.

**Fig. 9:**
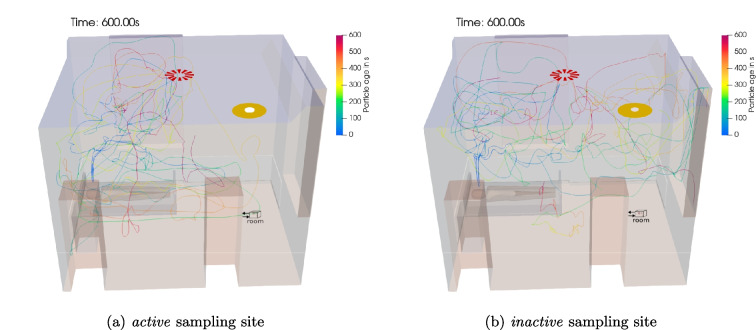
Particle pathlines while *singing* with active HVAC system and an **a** *active* and **b** *inactive* sampling device at the *room* position

The corresponding pathlines for 5 exemplary aerosol particles are displayed in Fig. 9. Here, the pathlines influenced by an *active* sampler show a vertical tendency near the patient and remain mostly in the bottom domain half near the *Outlet*, thus, preventing particles from escaping the domain. The pathlines with an *inactive* sampler show a significant concentration in the upper domain half near the *Outlet* explaining the increased amount of escaping particles compared to an active collector.

**Fig. 10:**
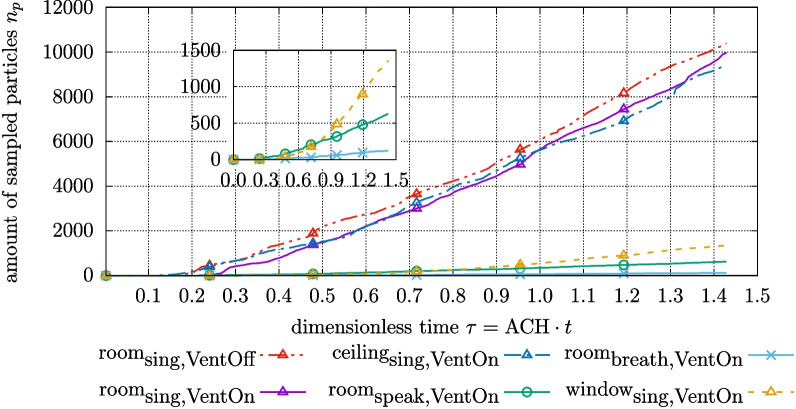
Amount of sampled particles $n_{p}$ over simulation time *t* for different sampler positions and exhalation types

In Fig. 10, the amount of sampled particles is displayed for different exhalation modes, collector positions, and ventilation settings. It can be seen, that the *room* and *ceiling* site plots with a *singing* patient are very similar across the investigated time frame despite the *room* site being further away from the droplet releasing patient.

However, the *window* site yields notably less particles compared to the *room* and *ceiling* sampling position under similar conditions. Moreover, the performance of the *window* site with a *singing* patient is in the range of the *room* sampler at much lower aerosol emission rates (*mouth breathing* and *speaking*). It is assumed, that the exhaled droplets evaporate shortly after being injected, thus, increasing affection by air flow with particles rarely sinking in the vicinity of the *window* site which renders this position inferior to the *room* and *ceiling* position for the investigated configurations.

In addition, deactivating the ventilation system leads to an increase in sampled aerosol particles as observed for the *room* collector and a *singing* patient.

## Discussion

Our findings highlight the interaction between the sampling device and the room air flow with the corresponding influence on the aerosol distribution within a single hospital patient room. In addition, a strong correlation between the collection results and the sampler location is observed.

Overall, the *ceiling* site proves to be in line with expectations for a sampler close and above the patient in the vicinity of the exhalation cone as observed by an air sampling campaign by Baig et al. (2022). In addition, it has to be noted that the emitted particles’ main presence is in the upper room half for the most investigated cases as seen by Li et al. (2021); Baig et al. (2022); Huang et al. (2022) and Karami et al. (2023). Therefore, such a sampling position seems to be an excellent choice to evaluate the infection risk at head height for a standing person.

However, the *room* site with the air exhaust helping to direct aerosol particles into the sampler’s working space could provide more comfort for the patient due to the increased distance to the patient while providing similar performance in terms of the sampled particle yield. This finding is contrary to Huang et al. (2022) where a 4-bed patient room is numerically investigated and the sampler in the middle of the room yields the lowest sampled particle amount. It must be mentioned that the samplers in Huang et al. (2022) do not include the inlet and exhaust which further indicates the importance of accurate sampler modeling to resolve aerosol distribution.

Furthermore, a counter-intuitive increase in aerosol particles remaining in the investigated systems is observed with *active* samplers, in particular the *room* sampling site. This along with the noticed sampler-induced particle circulation provides an explanation for the good sampler performance this far from the aerosol emitting source as observed in the literature. Such recirculation is also described by Xu et al. (2023) assuming to contribute towards an elevated infection risk.

Therefore, it can be concluded that the sampler has a major influence on the air flow and the corresponding aerosol distribution as implied by Zhang et al. (2019) and Saw et al. (2021), leading to positive viral findings in more remote places due to beneficial, sampler-induced air flow. However, this influence potentially can distort conclusions drawn from air sampling campaigns, thus, rendering preliminary numerical analysis of planned, large-scale collection campaigns advantageous to estimate the resulting impact or provide valuable insight for the sampler positioning to increase the sampling success rate.

Moreover, the findings on the influence of HVAC on the sampler performance suggest an increased collected particle yield with the investigated ventilation layout if the HVAC system is turned off, thus, further elevating the sampling success rate while increasing infection risk in the confined space as observed by Kim et al. (2023). Therefore, it is suggested to wait some time with an activated HVAC system after completion of the collection process until entering the patient’s room to reduce the infection risk of medical staff while increasing ACH in already well-ventilated areas is questionable according to Vita et al. (2023).

However, the ventilation layout can strongly influence the room air flow and therefore the aerosol distribution as observed by Baig et al. (2022) and Saw et al. (2021). Here, subsequent investigations with regards to different ventilation rates, due to the range of ACH in positive tested patient rooms (Wilson et al. 2022), multiple ventilation layouts (Baig et al. 2022; Saw et al. 2021; Karami et al. 2023) to further assess the influence on particle reduction and studies determining the necessary time for the infection risk to settle are needed.

## Conclusion

The interaction of air sampling devices, the room air flow within a single patient hospital room, and the corresponding aerosol concentration were numerically investigated in a 3D model using Lagrangian particles.

The findings of this study imply that the main aerosol concentration is present in the upper room half making sampling positions close and above the patient an effective choice.

Furthermore, a counter-intuitive increase in the aerosol concentration within the room is observed with *active* sampling devices which elevates the infection risk for medical personal during and shortly after the collection process. Therefore, it is recommended to wait some time with an active HVAC system after the sampling before entering the patient’s room.

This aerosol increase is caused by occurring aerosol recirculation induced by *active* samplers, thus, highlighting the strong interaction between room flow and sampler. Therefore, it has to be noted that conclusions drawn from sampling campaigns can be distorted. Large-scale air sampling campaigns could benefit from previously conducted numerical investigations to estimate the influence of sampling devices on the results and derive advantageous sampling sites.

Moreover, it was found that sampling positions in more remote locations can yield similar performance to samplers closer to the patient which could result in more comfort for the patient. However, ventilation layouts and settings have a major impact on the room flow necessitating subsequent studies quantifying these influences to determine more optimal sampling sites.

Additionally, the investigation of particle deposition along the airborne particle dispersion in future studies could further improve the prediction of contaminated areas for risk mitigation as well as surface collection campaigns conducted separately or in tandem with air sampling efforts.

Furthermore, a conclusive study of sampler volume inflow fluxes and collection time is advised in the scope of single hospital patient rooms to fully comprehend the interaction between sampling devices, room flow, and collection results to derive applicable guidelines for future sampling and monitoring campaigns in even more challenging environments.

## Data Availability

The datasets used and/or analyzed during the current study are available from the corresponding author on reasonable request.

## Footnotes

1 The numerical study was conceptualized and executed in tandem with an experimental virion sampling campaign in dedicated patient rooms in St. Georg Hospital, Leipzig. Physicians from the University of Leipzig Medical Center and St. Georg Hospital conducted the sampling campaign with the Coriolis sampler for due to prior experience and availability of the device. Our numerical investigation was designed to aid the sampling campaign by preliminary simulations suggesting effective sampling positions. Unfortunately, the sampling campaign could not sample virions above the expected detection limit of around 1000 virions.

2 As part of the patient study at St. Georg Hospital, the medical team also investigated the activity of “singing” as a worst-case scenario. Accordingly, the corresponding aerosol emissions were analyzed within the framework of our numerical study.

## Code availability

Source code is available upon reasonable request.
